# Molecular characterization analysis of PANoptosis-related genes in colorectal cancer based on bioinformatic analysis

**DOI:** 10.1371/journal.pone.0307651

**Published:** 2024-08-26

**Authors:** Mengyang Zhang, Wen Li, Yubo Zhao, Ling Qi, Yonglong Xiao, Donglian Liu, TieLi Peng

**Affiliations:** 1 Division of Gastroenterology, Institute of Digestive Disease, the Affiliated Qingyuan Hospital (Qingyuan People's Hospital), Guangzhou Medical University, Guang Dong, China; 2 College of Pharmacy, Dali University, Yunnan, China; 3 Institute of Digestive Disease, the Affiliated Qingyuan Hospital (Qingyuan People's Hospital), Guangzhou Medical University, Guang Dong, China; Huashan Hospital Fudan University, CHINA

## Abstract

Colorectal cancer (CRC) ranks as the third most prevalent cancer globally and stands as the second principal contributor to cancer-related fatalities. Recently, emerging research has emphasized the role of pan apoptosis (PANoptosis) in tumor development and anti-tumor therapy. In the course of this investigation, we meticulously identified and conducted a correlation analysis between differentially expressed genes associated with PANoptosis in CRC (CPAN_DEGs) and the proportion of immune cells. Subsequently, we formulated a prognostic score based on the CPAN_DEGs. Further our analysis revealed a noteworthy reduction in UNC5D mRNA expression within HCT116, HT29 and SW480 cells, as validated by qRT-PCR assay. Furthermore, scrutinizing the TCGA database unveiled a distinctive trend wherein individuals with the low UNC5D expression exhibited significantly reduced overall survival compared to their counterparts with the high UNC5D levels. The drug susceptibility analysis of UNC5D was further performed, which showed that UNC5D was corassociated with the sensitivity of CRC to 6-Thioguanine. The outcomes of our investigation underscore the mechanisms by which PANoptosis influences immune dysregulation as well as prognostic outcome in CRC.

## Introduction

CRC stands as the third most prevalent malignancy globally and ranks second in cancer-related mortality [1, 2]. Despite the endorsement of oxaliplatin based chemotherapy as the standard therapeutic approach, the emergence of chemotherapy resistance can lead to recurrence and metastasis, resulting in poor prognosis for colorectal cancer patients and urgent need to be tackled. The pathogenic landscape of colorectal cancer encompasses genetic and epigenetic alterations, metabolic perturbations, and aberrant signaling pathways [3–6]. Therefore, the imperative lies in unraveling the intricate molecular mechanisms underpinning CRC and discerning precise predictive biomarkers. Such endeavors are crucial for early diagnosis and personalized treatment of CRC. And they play a vital role in vigilantly monitoring the trajectory of cancer recurrence. Furthermore, they are essential for keeping a close eye on the progression of metastasis in cancer.

More and more research delves into the intricate mechanisms governing cellular demise across a spectrum of diseases. The comprehensive exploration of programmed cell death (PCD) pathways, encompassing cellular pyroptosis, apoptosis, necroptosis, and ferroptosis, stands as a focal point in research addressing the intricate facets of inflammation and autoimmune diseases [7]. In 2019, Professor Kanneganti’s team proposed an emerging concept, pan apoptosis (PANoptosis) [8]. PANoptosis, is an inflammatory programmed cell death characterized by several pivotal features [9–12]. The initiation of PANoptosis involves the regulatory interplay of ZBP1, pyrin, and AIM2, culminating in the facilitation of PANoptosome formation mediated by AIM2 [13]. PANoptosomes comprise diverse inflammasome sensors, like NLRP3, etc. along as well as key regulatory factors for cell death, like caspase-1, etc. in cell pyroptosis, FADD in cell apoptosis, and RIPK1, etc. in necroptosis [13]. The phenomenon of PANoptosis has been documented across a spectrum of infectious and neoplastic maladies, intricately entwined with the processes of inflammation and the activation of the immune response [14, 15].

Recently, emerging research has emphasized the role of PANoptosis in tumor development and anti-tumor therapy. IRF1, TNF-α, and IFN-γ have been shown to induce PAN apoptosis to prevent tumorigenesis associated with CRC colitis [15]. The latest studies suggest that phosphorylated NFS1 weakens oxaliplatin-based chemosensitivity of colorectal cancer by preventing PANoptosis [9]. The induction of systemic inflammation through the liberation of pro-inflammatory intracellular contents positions PANoptosis as a promising approach in the realm of solid tumor immunotherapy [16]. Therefore, delving deeper into the potential mechanisms of PANoptosis offers novel prospects for the formulation of efficacious strategies in CRC immunotherapy. The primary objective of this investigation is to discern the distinctive expression patterns of PANoptosis-associated genes in CRC through bioinformatics analysis, and to explore the predictive accuracy of key PANoptosis-associated genes for CRC prognosis and their clinical potential for personalized decision-making in CRC immunotherapy. We identified the novel molecular signatures of PANoptosis-related genes, which may serve as a potential diagnostic model for CRC. And this study highlights the potential role of UNC5D and helps in future discovery of novel pathways targeting PANoptosis for CRC.

## Methods

### Identification of the PANoptosis-associated differentially expressed genes for CRC

In the course of our investigation, we conducted differential analysis utilizing the "limma" package (version 3.40.6). This analytical approach aimed to discern the distinctive expression profiles of genes, thereby identifying differentially expressed genes between the normal and cancer groups The data utilized for this analysis were sourced from Cancer Genome Atlas (TCGA) database regarding colon adenocarcinoma (COAD) and rectal adenocarcinoma (READ). Stringent criteria were applied for the selection of DEGs, with a threshold set at *P*<0.05 and an absolute |log2FC|>1.5. In order to generate a list of PANoptosis genes, we used the gene lists of cell pyroptosis, apoptosis, necroptosis, and ferroptosis that were merged from two previous articles [16, 17], while eliminating any redundant genes to compile a single gene list and incorporating it into subsequent analysis (S1 Table in S1 File).

### GO and KEGG enrichment analysis

Statistical analyses and visualizations were executed utilizing the software R, with the ggplot2 package employed for graphical representations. For in-depth functional insights, the clusterProfiler package facilitated the execution of GO and KEGG analyses. To augment the exploration of functional mechanisms, the Metascape database (accessible at https://metascape.org/) was additionally employed.

### Construction and identification of the hub gene models

Acquiring the STAR counts data and clinical details for CRC from the TCGA dataset, we proceeded to extract TPM format data. Following extraction, a log2 normalization (TPM+1) was applied, meticulously preserving samples inclusive of RNAseq data and clinical information for subsequent analytical endeavors. The inclusion criteria involved the meticulous retention of samples possessing both RNAseq data and clinical information for ensuing analyses. In the realm of survival analysis, log-rank tests were leveraged to discern potential survival disparities across two or more specified groups in KM survival analysis, and to perform timeROC analysis to determine the accuracy of the predictive model. Feature selection, a critical component, was executed using the LASSO regression algorithm via the glmnet package in R software, incorporating a 10-fold cross-validation approach for robustness.

### Analysis of immune cell infiltration

Utilizing the R software package pheatmap, we visually depicted immune cell infiltration. For the assessment of the correlation between quantitative variables devoid of a normal distribution, Spearman’s correlation analysis was employed. Statistical significance was defined at a *P-*value threshold below 0.05.

### Correlation analysis between CPAN_DEGs and immune score

STAR-counts data and clinical information for colorectal cancer were downloaded from the TCGA database (https://portal.gdc.com). Converting counts data to TPM and normalizing the data log2 (TPM+1), retaining samples with RNAseq data and clinical information for subsequent analysis. For a reliable immune score evaluation, we used the R software package: immunedeconv. Analysis and visualization were performed using the ggClusterNet package. All of the above analytical methods and R software packages were performed using the R software. A *p* <0.05 was considered statistically significant.

### Construction of the prognostic signature of CPAN_DEGs in CRC

The Lasso (Least absolute shrinkage and selection operator) method is a compressed estimate. It obtains a more refined model by constructing a penalty function, so that it compresses some coefficients and sets some ones to zero. Therefore, the advantage of subset shrinkage is preserved, which is a kind of biased estimation with complex collinearity data, which can realize the selection of variables while parameter estimation, and better solve the multicollinearity problem in regression analysis, when the lambda minimum model reaches the optimum. A larger AUC value and a smaller Log-rank p values indicate a better prediction. In the model selection, we extracted the number of genes with the AUC results of the survivalROC run above 0.7 for the first time, and selected the gene model with the maximum AUC value if none were above 0.7. STAR-counts data and clinical information for colorectal cancer were downloaded from the TCGA database (https://portal.gdc.com). Converting counts data to TPM and normalizing the data log2 (TPM+1), retaining samples with RNAseq data and clinical information for subsequent analysis. Log rank was used to test the survival difference of KM survival analysis between the two or more groups, and the timeROC analysis was comparing the accuracy of the prediction model. Feature selection was performed using the glmnet package using the Least absolute shrinkage and selection operator regression algorithm, 10-fold cross-validation, and multivariate Cox regression analysis using the survival package. The multifactor Cox regression analysis, and the optimal model is selected as the final model. For the Kaplan-Meier curve, the *p*-value and the hazard ratio (HR) with a 95% confidence interval (CI) were obtained by logrank-test and univariate Cox regression. All of the above analytical methods and R software packages were performed using the R software. A *p* <0.05 was considered statistically significant.

### Drug sensitivity of UNC5D

The NCI-60 compound activity and RNA-seq data were downloaded from the cellminer database (https://discover.nci.nih.gov/cellminer/home.do) [18]. Then the correlation between UNC5D gene expression and drug IC50 values were analyzed. “impute”, “limma”, “ggplot2”, and “ggpubr” package in R software were used.

### Real-time PCR (qRT-PCR) assay

Cellular RNA extraction from NCM460, HCT116, HT29 and SW480 cells was executed using Trizol reagent. Evaluation of *UNC5D* mRNA expression levels was performed through the One-Step SYBR PrimeScript primary RT-PCR kit from Takara Bio, Beijing, China, with the 7500 Fast RT-PCR system overseeing the reaction. GAPDH served as the designated endogenous control throughout the experiment. S2 Table in S1 File delineates the primer sequences employed in this process.

### Statistical analysis

Analysis of experimental data was conducted employing the GraphPad Prism statistical software. Group distinctions were evaluated through the application of either a Student’s t-test, or a one-way ANOVA, employing rigorous statistical methodologies for comparative analysis. Presentation of the data involved expressing results as the mean ± standard deviation (SD). Software R was enlisted for both data visualization and statistical analysis, with the R Package "ggplot2" facilitating graphical representations. Statistical significance was attributed to *P*-values below 0.05.

## Results

### Identification of PANoptosis-associated differentially expressed genes for CRC

**Fig 1:**
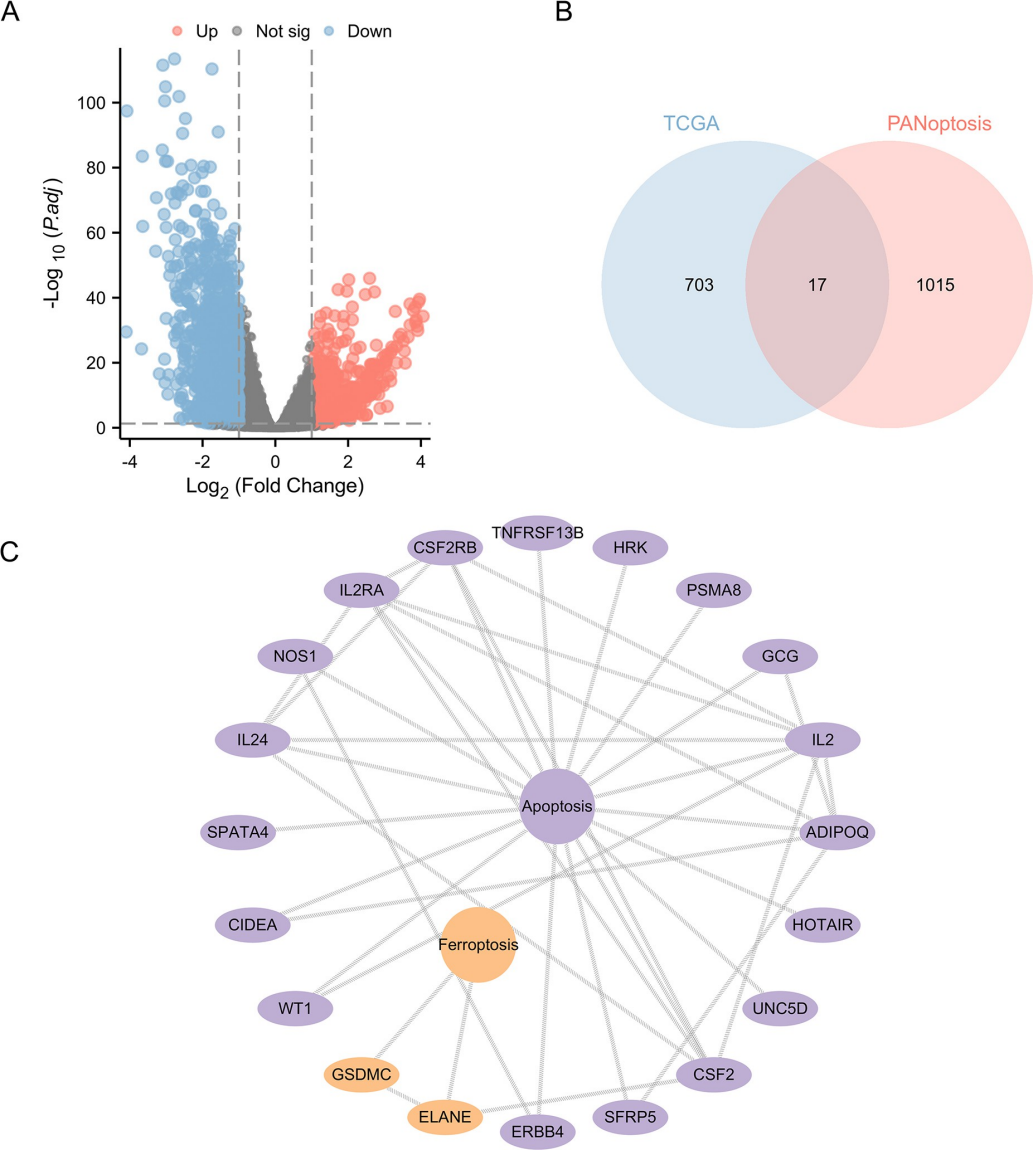
Identification of PANoptosis-associated differentially expressed genes for CRC **(A)** Volcano plot showed the differential genes of in the TCGA database. **(B)** The Venn diagram about the differential genes of TCGA and the PANoptosis related dataset. **(C)** The CPAN_DEGs list.

We first downloaded and analyzed the genes differentially expressed in CRC in the TCGA database (Fig 1A). Comprising the PANoptosis gene set were apoptosis (1001 genes), necroptosis (16 genes), pyroptosis (46 genes) and ferroptosis (13 genes). We drew Venn diagrams of the TCGA and PANoptosis gene sets and identified 17 PANoptosis-associated differentially expressed genes for CRC (CPAN_DEGs) (Fig 1B). The CPAN_DEGs consisted of apoptosis (15 genes) and ferroptosis (2 genes), and Fig 1C showed the CPAN_DEGs list.

### Functional enrichment analysis of PANoptosis-associated differentially expressed genes in CRC

**Fig 2:**
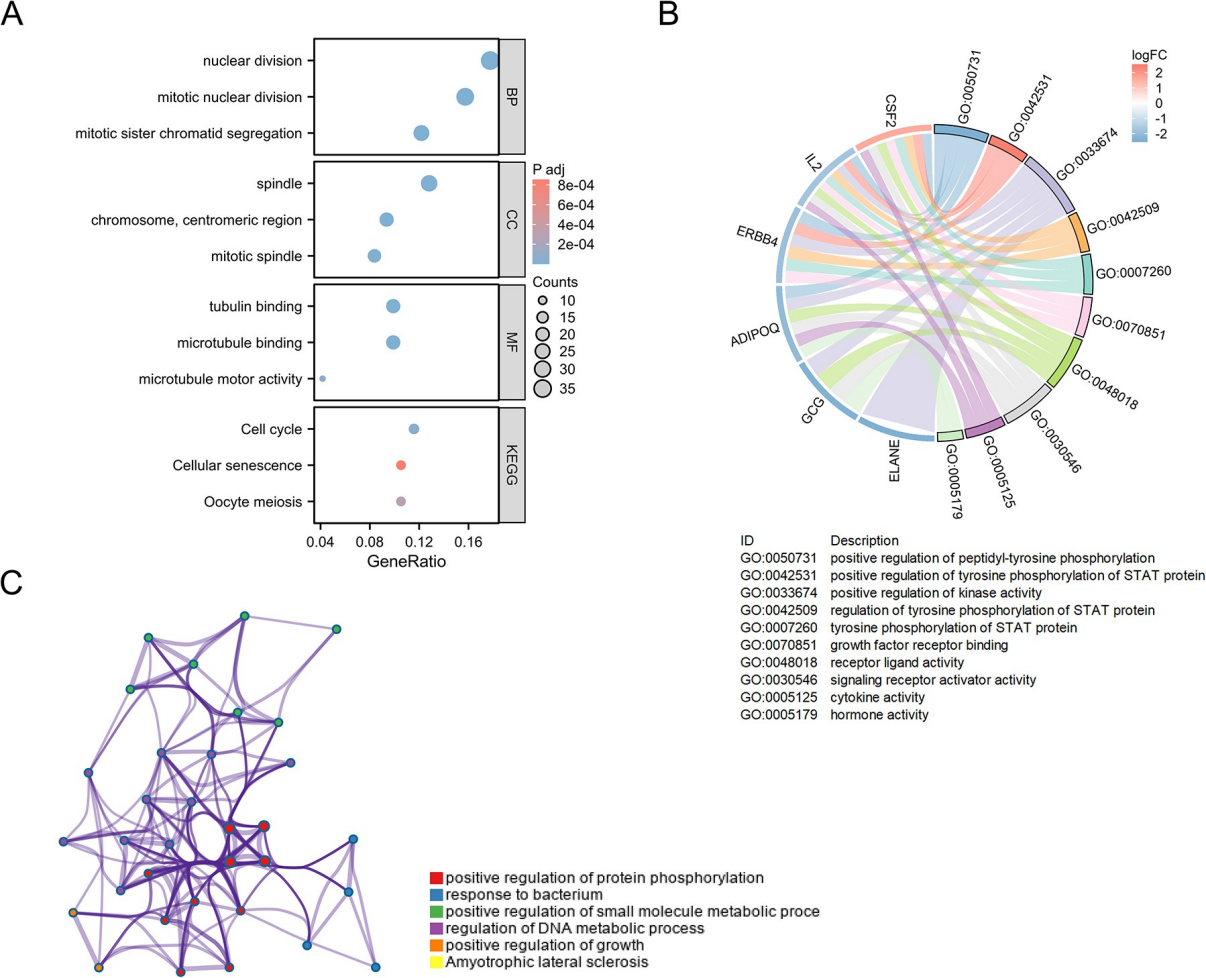
Functional Enrichment Analysis of PANoptosis-associated differentially expressed genes in CRC **(A)** The enriched item in the GO, KEGG analysis. **(B)** Chord Diagram displaying the functional enrichment analysis of the CPAN_DEGs. **(C)** Metascape enrichment networks showing the intra-cluster and intercluster similarities of enriched terms, colour code reflecting cluster annotations.

Subsequent to this, an in-depth analysis of the biological functions and associated signaling pathways of CPAN_DEGs was undertaken through the application of GO and KEGG analytical approaches. Biological Process (BP) analysis underscores the predominant enrichment of CPAN_DEGs in processes pivotal for cellular functionality, including nuclear division, mitotic nuclear division, and mitotic sister chromatid segregation. Cell Component (CC) analysis a distinct enrichment profile emerges, positioning CPAN_DEGs predominantly within cellular structures such as the spindle, chromosome, centromeric region, and mitotic spindle. Molecular Function (MF) analysis the focus sharpens on functional enrichments related to tubulin binding, microtubule binding, and microtubule motor activity which can provide a comprehensive understanding of the intricate roles and localizations governed by CPAN_DEGs within cellular processes. In the realm of KEGG pathway enrichment analysis, it was discerned that the predominant associations of CPAN_DEGs were aligned with processes integral to the Cell cycle, Cellular senescence and Oocyte meiosis (Fig 2A). We further perform GOKEGG combined with logFC enrichment analysis, and Fig 2B shows that CSF2, IL2, ERBB4, ADIPOQ, GCG and ELANE were mainly enriched in positive regulation of peptidyl-tyrosine phosphorylation, positive regulation of tyrosine phosphorylation of STAT protein, positive regulation of kinase activity, regulation of tyrosine phosphorylation of STAT protein, tyrosine phosphorylation of STAT protein, growth factor receptor binding, receptor ligand activity, signaling receptor activator activity, cytokine activity and hormone activity. Further using the Metascape database analyzed enriched ontology clusters (Fig 2C).

### Correlation between expression of CPAN_DEGs and immune infiltration levels in CRC

**Fig 3:**
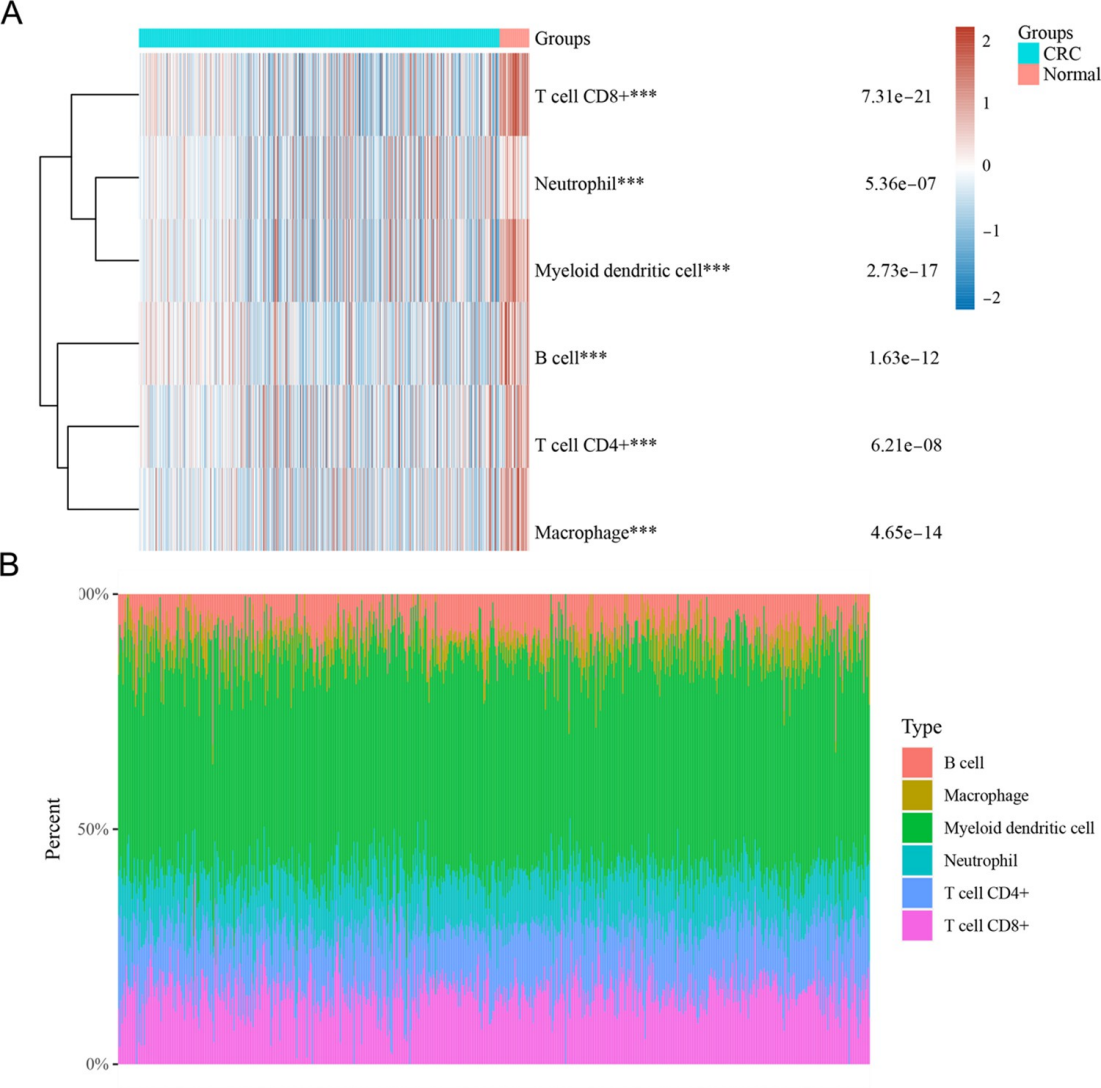
Landscape of immune in CRC **(A)** Immune cell score heatmap, different colors represent different expression distribution in different samples. **(B)** The percentage abundance of tumor infiltrating immune cells in each sample.

**Fig 4:**
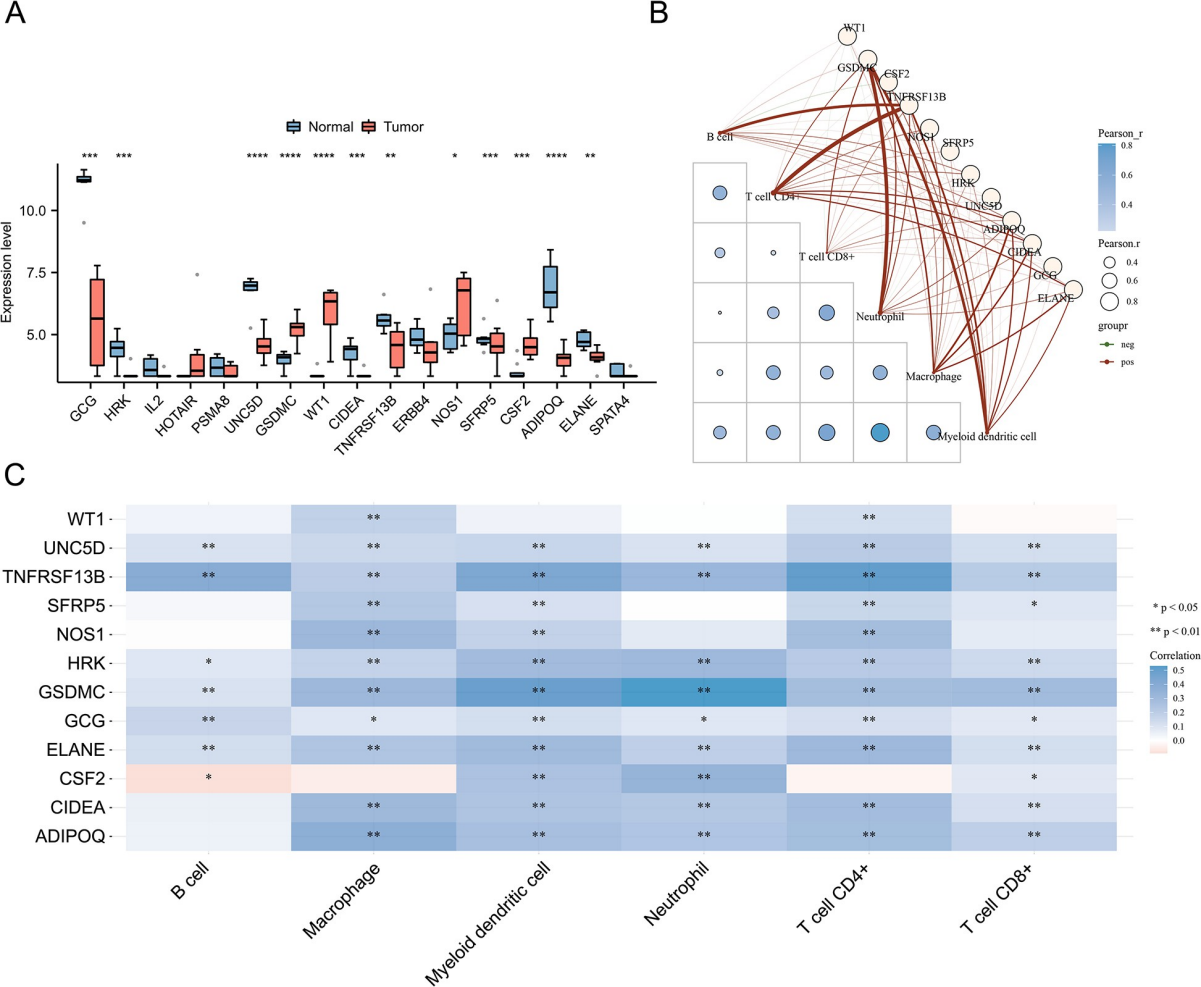
Correlation between Expression of CPAN_DEGs and Immune Infiltration Levels in CRC **(A)** The expression of 17 CPAN_DEGs in CRC and normal tissues of GSE138202 dataset. **(B)** The heat map in the schematic represents the correlation analysis of the immune score itself; the red line in the schematic represents the negative correlation between the gene expression and the immune score. **(C)** A heatmap of the correlation between multiple genes and immune score (blue represents positive correlation whereas red represents negative correlation), ** for *p* < 0.01, * for *p* < 0.05.

Conducting a comprehensive investigation into the immune cell landscape, our analysis revealed markedly elevated proportions of B cells, Macrophages, Myeloid dendritic cells, Neutrophils, T cell CD4+, and T cell CD8+ in patients with CRC compared to those in the normal control cohort (Fig 3A and 3B). We next analyzed the expression of CPAN _ DEGs in CRC compared to normal tissues, Fig 4A showed that WT1, GSDMC, CSF2 and NOS1 emerged as significantly elevated in the tumors than in normal tissues, and TNFRSF13B, HRK, UNC5D, ADIPOQ, CIDEA, GCG and ELANE expression were significantly reduced. Building upon this foundation, an exhaustive correlation analysis was executed between the identified CPAN_DEGs and the proportions of immune cells. The outcomes unveiled a spectrum of associations: WT1 expression displayed a negative correlation with immune infiltration levels of Macrophages and T cell CD4+. Concurrently, UNC5D, TNFRSF13B, HRK, GSDMC, GCG, and ELANE exhibited negative associations with immune infiltration levels of B cells, Macrophages, Myeloid dendritic cells, Neutrophils, T cell CD4+, and T cell CD8+. SFRP5 displayed a negative correlation with immune infiltration levels of Macrophages, Myeloid dendritic cells, T cell CD4+, and T cell CD8+. Furthermore, NOS1 exhibited a negative association with immune infiltration levels of Macrophages, Myeloid dendritic cells, and T cell CD4+. CSF2 displayed a negative correlation with immune infiltration levels of B cells, Myeloid dendritic cells, Neutrophils, and T cell CD8+. Finally, CIDEA and ADIPOQ demonstrated negative associations with immune infiltration levels of Macrophages, Myeloid dendritic cells, Neutrophils, T cell CD4+, and T cell CD8+ (Fig 4B and 4C).

### Construction of the prognostic signature of CPAN_DEGs in CRC

**Fig 5:**
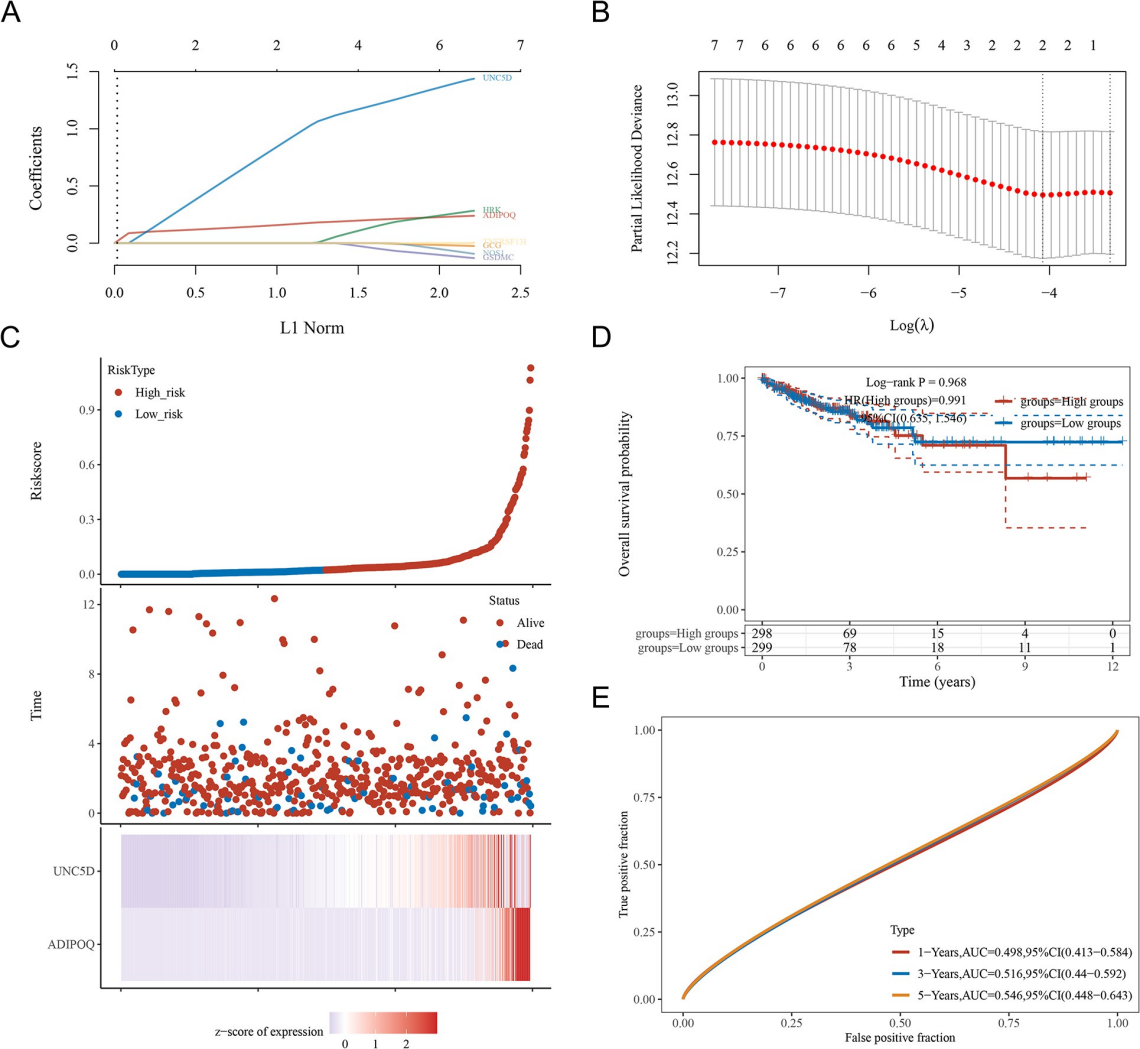
Construction of the Prognostic Signature of CPAN_DEGs in CRC (**A**) LASSO coefficient profiles of CPAN_DEGs in CRC. A coefficient profile plot was generated against the log (lambda) sequence. (**B**) Selection of the optimal parameter (lambda) in the LASSO model for CRC. (**C**) Distribution of risk score, survival status and the expression of prognostic CPAN_DEGs. (**D**) Kaplan-Meier survival analysis of the CPAN_DEGs signature from TCGA dataset, comparison among different groups was made by log-rank test. (**E**) The ROC curve of CPAN_DEGs. The higher values of AUC corresponding to higher predictive power.

Prognostic gene signatures derived from CPAN_DEG were meticulously established through the implementation of LASSO regression analysis, as visually represented (Fig 5A and 5B). In delineating disease specific survival outcomes for CRC patients, a discerning 2-gene signature was identified to formulate the prognostic score. This score, calculated with the respective regression coefficients, takes the form of Riskscore = (0.1341) * ADIPOQ + (0.5794) * UNC5D. The intricate relationships among risk scores, survival time, and survival status within the selected datasets are vividly depicted (Fig 5C and 5D). Evaluation of the predictive value of the OS risk score was accomplished through ROC curves, yielding prediction accuracies of 0.498, 0.516, and 0.546 for the 1-year, 3-year, and 5-year ROC curves, respectively (Fig 5E).

### Related drug analysis of UNC5D

**Fig 6:**
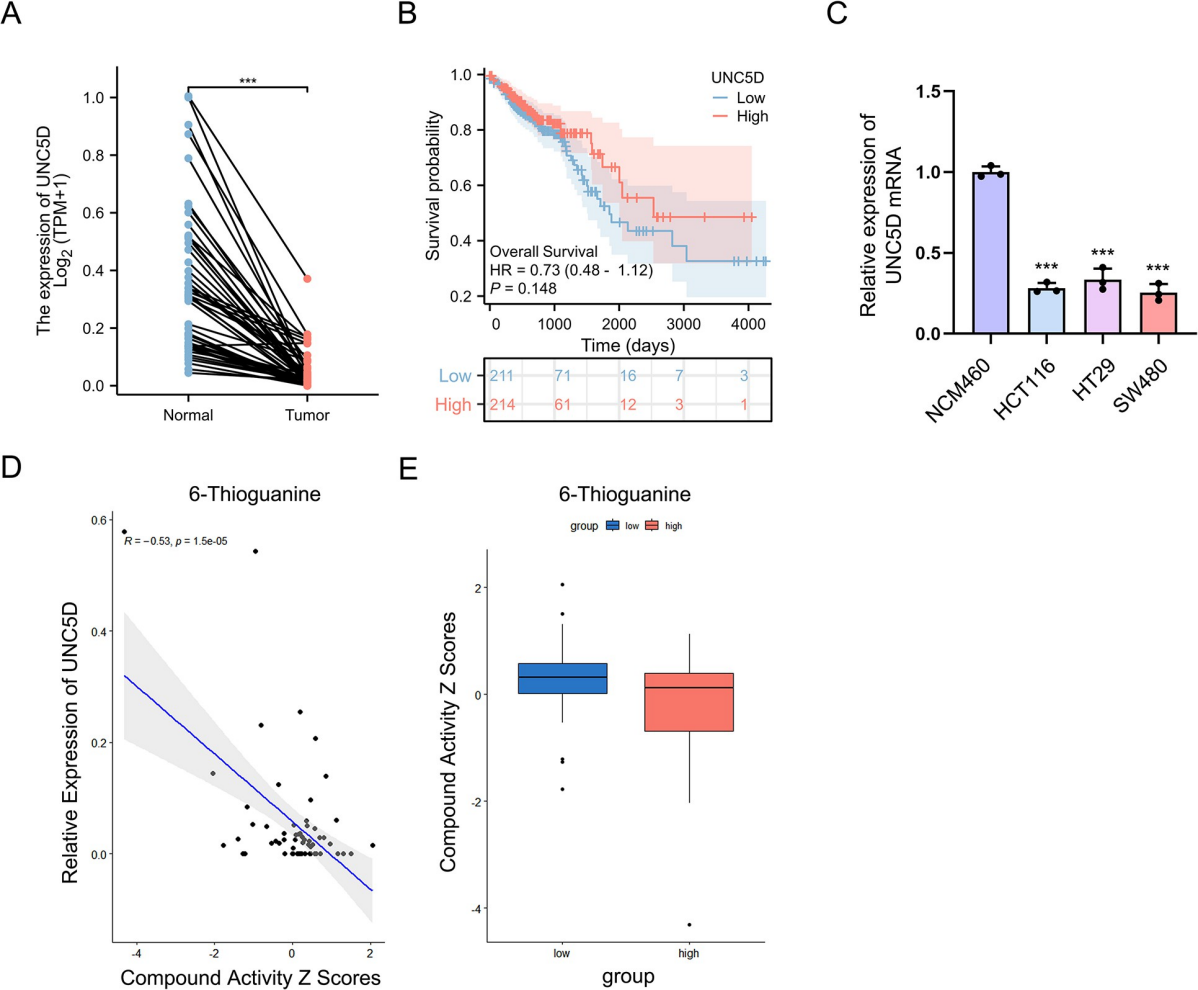
Related Drug Analysis of UNC5D (A) The expression level of UNC5D in CRC tissue and adjacent tissues. (B) Kaplan-Meier OS analysis of UNC5D expression in patients with CRC. (C) qRT-PCR employed for the detection of the UNC5D mRNA expression level in NCM460, HCT116, HT29 and SW480 cells. Each value represents the mean ± SD (n = 3, each group), **P<0.01, compared with NCM460 group. (D, E) Drug with the most significant correlation with UNC5D in CellMiner database.

**Fig 7:**
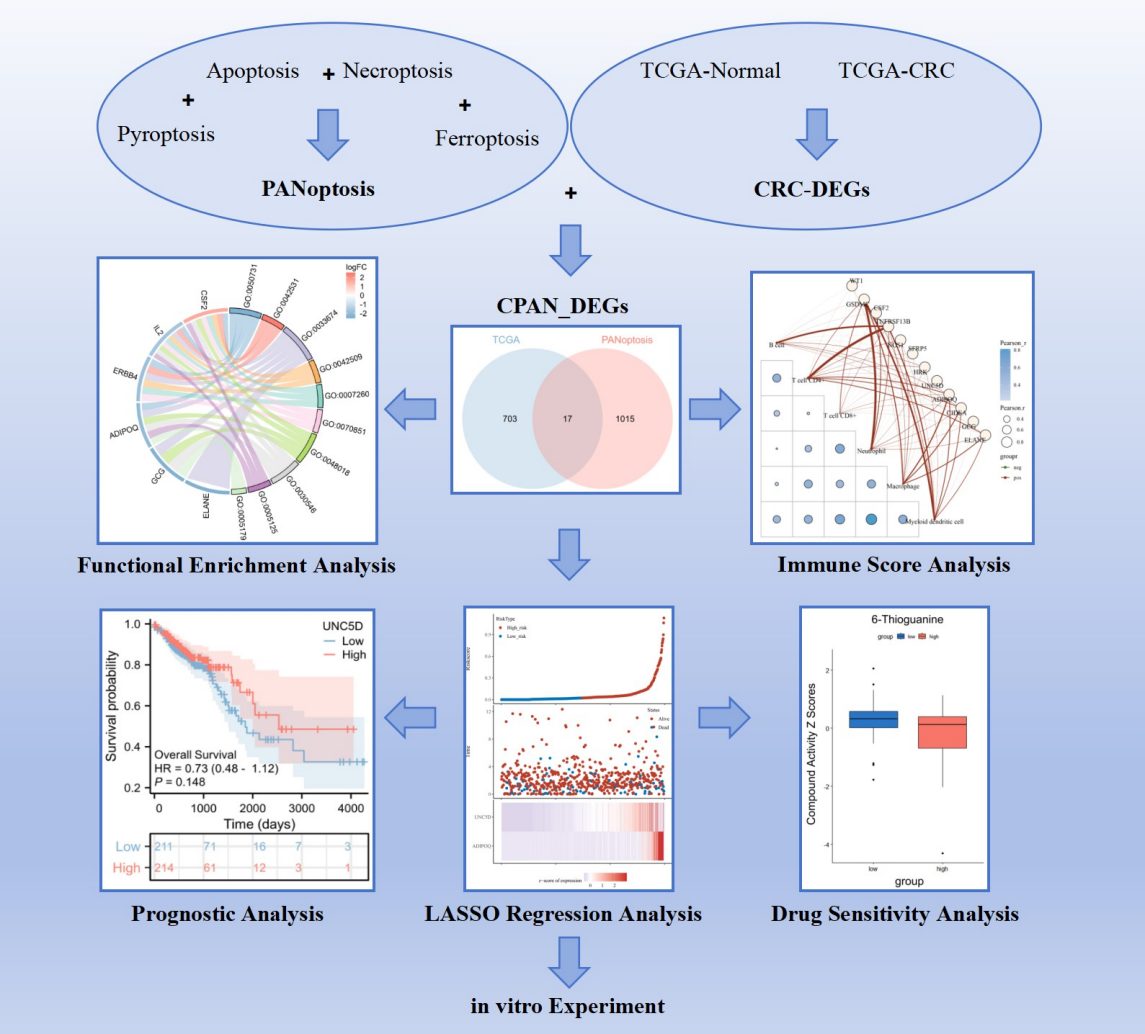
The brief flow chart of the study.

We further performed expression and survival analysis of UNC5D in CRC by TCGA database, the findings indicated that UNC5D was lowly expressed in CRC (Fig 6A), and patients with low UNC5D had lower overall survival than the high UNC5D group patients (Fig 6B). Subsequently, qRT-PCR assays were conducted on three colorectal cancer cell lines, namely HCT116, HT29 and SW480 cells, revealing a notable reduction in UNC5D mRNA expression levels in all three cell lines (Fig 6C). To assess the potential impact of UNC5D on drug response, we executed Pearson correlation analysis to quantify the relationship between UNC5D gene expression and compound sensitivity. Retrieving data from the CellMiner database (available at https://discover.nci.nih.gov/cellminer/), we obtained the mRNA expression profiles and drug activity information pertaining to the UNC5D gene. The analysis revealed a negative correlation between UNC5D and 6-Thioguanine (Fig 6D and 6E). A brief flow chart of this study is shown in Fig 7.

## Discussion

In the past decade, immunotherapy has attracted great attention due to its successful long-term and persistent response in solid tumors that have historically posed treatment challenges. PANoptosis is a key inflammatory PCD pathway with intricate features encompassing pyroptosis, cell apoptosis, and necroptosis, defying singular classification under any of these three PCD pathways [19]. The induction of systemic inflammation through the release of pro-inflammatory intracellular contents positions PANoptosis as a prospective immunotherapeutic avenue for addressing solid tumors [20]. Therefore, a more profound exploration into the potential mechanisms governing PANoptosis unveils novel prospects for the formulation of effective strategies in the realm of CRC immunotherapy.

Inflammation plays a major role in colitis-associated colorectal cancer. The inflammasome is a key component of the inflammatory response [21]. In addition to the induction of pyroptotic cell death by the inflammasome, the inflammasome can function as components of the apoptotic body, a multifaceted macromolecular complex that regulates apoptosis. PANoptosis includes redundancy and crosstalk between components of the cell death pathway and cannot be explained solely by pyroptosis, apoptosis or necrotizing apoptosis [13, 22]. Studies have shown that inflammasome plays a role in colorectal tumorigenesis [23]. Studies have shown that IRF1 can prevent colorectal cancer by regulating PANoptosis [15]. NFS1 deficiency can trigger PANoptosis and shows synergistic effects by increasing ROS levels with oxaliplatin treatment, and NFS1 inhibition is a promising strategy to improve the outcome of platinum-based chemotherapy in CRC [9]. Therefore, studying the role of PANoptosis in the pathogenesis of colorectal cancer can provide new ideas for the treatment and prevention of colorectal tumors, and targeting PANoptosis has the potential for future use in colorectal cancer treatment.

Apoptosis is one of the key mechanisms of drug resistance in tumor cells [24]. UNC5D, the fourth constituent of the human UNC5 receptor family, interfaces with the shared ligand netrin-1, akin to its counterparts within the UNC5 family and the tumor suppressor gene DCC [25, 26]. Characterized by the presence of the intracellular Zo-1-like (ZU5) domain, the death domain (DD), and potential caspase cleavage sites, the UNC5 family collectively encompasses critical structural elements [27]. Each member of the UNC5 family induces cell death through distinctive mechanisms. UNC5A orchestrates apoptotic cell death through its ZU5 domain interaction with NRAGE [28], whereas UNC5B employs its DD to recruit DAPK when inducing cell apoptosis [29]. Among the members of the UNC5 family, only UNC5D has an inducing effect on NGF depletion. Studies have shown that PCD induced by NGF deficiency in NGF-dependent human neuroblastoma cells, UNC5D emerges as a key player in this intricate regulatory network [30]. High levels of UNC5D mRNA expression exhibit a statistically significant correlation with good prognosis among neuroblastoma patients. Other studies have shown that UNC5D plays a pivotal role in governing p53-dependent cell apoptosis within neuroblastoma cells [31] Moreover, it exhibits downregulation in bladder cancer, potentially influencing cisplatin-induced apoptosis in bladder cancer cells [32]. In the context of prostate cancer, UNC5D exerts its influence by inhibiting cell metastasis through the activation of death-related protein kinase 1 [33]. However, the regulation of CRC by UNC5D has not been fully studied yet.

Within the intricate framework of physiological homeostasis, cell death emerges as a pivotal and indispensable process, contributing significantly to the intricate balance inherent in the body. More and more evidence suggests that pathological triggers simultaneously activate embracing the entirety of cell death pathways, this fosters the evolution and conceptualization of the PANoptosis paradigm [34]. In the scope of this current study, we identified 17 PANoptosis-associated differentially expressed genes for CRC through bioinformatic analysis. We performed a correlation analysis between these CPAN_DEGs and the proportion of immune cells, the expression level of UNC5D, TNFRSF13B, HRK, GSDMC, GCG, and ELANE demonstrated an inverse correlation with the immune infiltration level of B cells, Macrophage, Myeloid dendritic cell, Neutrophil, T cell CD4+ and T cell CD8+. Then we constructed the CPAN_DEGs-related prognostic score. Further, we found that UNC5D mRNA expression experienced a noteworthy reduction in HCT116, HT29 and SW480 cells by qRT-PCR assay. Furthermore, an examination of the TCGA database unveiled a discernible trend wherein individuals with low UNC5D expression exhibited significantly reduced overall survival compared to their counterparts within the high UNC5D expression cohort. 6-thioguanine (6-TG) is a synthetic guanosine analog with antitumor and immunosuppressive activities [35]. 6-TG treatment remodels the tumor microenvironment by increasing T and NK immune cells, making the tumor more sensitive to immune checkpoint blockade [36]. Studies have shown that treatment with the anticancer drug 6-TG increases the mismatch repair deficiency fraction and improves immune surveillance in CRC [37]. Human DNA mismatch repair (MMR) is involved in the response to certain chemotherapeutic agents, including 6-TG. Consistently, MMR deficient human tumor cells exhibited resistance to 6-TG damage, manifested by reduced G2-M arrest and reduced apoptosis [38]. The role of the BRCA1 (BReast-CAncer susceptibility gene 1) protein in regulating the 6-TG-induced MMR damage response: BRCA1 is a tumor suppressor gene involved in a variety of key cellular processes contributing to DNA repair and transcriptional regulation in response to DNA damage. BRCA1 mutations have been shown to cause genomic instability associated with G2-M checkpoint defects in DNA mismatch activation and BRCA1 positive cells are also more sensitive to 6-TG than BRCA1 mutant cells [39]. UNC5D is transcriptionally regulated by the tumor suppressor p53 and participates in p53-dependent apoptosis in response to DNA damage induced by conventional chemotherapeutic agents such as doxorubicin [25]. Therefore, it is speculated that the response of UNC5D to DNA damage may be the potential reason for its effect on 6-TG sensitivity, and the specific mechanism still needs to be further explored through molecular biology experiments. The drug susceptibility analysis of UNC5D was further performed, which showed that UNC5D was corassociated with the sensitivity of CRC to 6-TG. Our discoveries underscore the plausible engagement of PANoptosis in the intricacies of prognosis and immune dysregulation within the context of CRC.

There are potential confounding factors in the TCGA database analysis, for example, major gene expression differences were observed between Black and white patients in breast cancer, but can be significantly reduced or eliminated after adjusting for molecular subtypes [40, 41]. Treatment effect is also a potential confounder and should be considered and adjusted appropriately for when available. When confounding factors are present but are excluded from the model, the bias may manifest as an overestimate or underestimate of the true effect.In addition, the annotation of sample-related patient outcomes and treatment data obtained by TCGA is incomplete due to their relatively short clinical follow-up interval [40, 42].

## Supporting information

S1 File Containing with the following: S1 Table. A list of PANoptosis genes. **S2 Table.** Primers.
(DOCX)

S2 File Raw data file of qRT PCR. (XLSX)

S3 File The correlation between UNC5D expression and CRC sensitivity to 6-Thioguanine. (CSV)

S4 File The relevant data files of the database designed in the research. (XLSX)
