# Supplementary material for: Molecular characterization analysis of PANoptosis-related genes in colorectal cancer based on bioinformatic analysis
Source: PLoS One. 2024 Aug 26;19(8):e0307651. doi: 10.1371/journal.pone.0307651 (PMC11346968; doi:10.1371/journal.pone.0307651)
Supplement: S1 File — A list of PANoptosis genes. S2 Table. Primers. (DOCX) [file pone.0307651.s001.docx]

**Supplementary Table**

**Supplementary Table 1**

| Apoptosis Gene Symbol | Ferroptosis Gene Symbol | Ferroptosis Gene Symbol | Pyroptosis Gene Symbol |
| --- | --- | --- | --- |
| CASP8 | AIFM2 | RIPK1 | GSDMD |
| BCL2 | GPX4 | RIPK3 | GSDME |
| CASP3 | SLC7A11 | MLKL | NLRP3 |
| FAS | TP53 | ZBP1 | CASP1 |
| BAX | NFE2L2 | CASP8 | CASP4 |
| XIAP | ACSL4 | TNF | GSDMC |
| CFLAR | TFRC | CYLD | GSDMB |
| FASLG | NCOA4 | ITPK1 | NLRP1 |
| CASP10 | HMOX1 | IPMK | IL1B |
| TP53 | SLC40A1 | MAP3K7 | GZMB |
| AIFM1 | SLC3A2 | CASP6 | GSDMA |
| CASP9 | ALOX15 | TRPM7 | AIM2 |
| TNFSF10 | IREB2 | FAS | CARD8 |
| MCL1 |  | FASLG | CASP8 |
| BCL2L1 |  | TLR3 | GZMA |
| CYCS |  | FADD | PYCARD |
| BIRC5 |  |  | DPP9 |
| BCL2L11 |  |  | CASP5 |
| CASP7 |  |  | IL18 |
| TNFRSF10B |  |  | ZBP1 |
| PRKCD |  |  | CASP3 |
| CASP2 |  |  | NLRC4 |
| NAIP |  |  | DPP8 |
| TNF |  |  | MIR223 |
| BOK |  |  | CASP6 |
| CASP6 |  |  | TRIM24 |
| CASP1 |  |  | NAIP |
| MAPK8 |  |  | HMGB1 |
| PAWR |  |  | MEFV |
| AKT1 |  |  | [BAK1](http://ensembl.org/Homo_sapiens/Gene/Summary?db=core;g=BAK1) |
| BIRC2 |  |  | [TP63](http://ensembl.org/Homo_sapiens/Gene/Summary?db=core;g=TP63) |
| TNFRSF10A |  |  | [CHMP2B](http://ensembl.org/Homo_sapiens/Gene/Summary?db=core;g=CHMP2B) |
| PARP1 |  |  | [BAX](http://ensembl.org/Homo_sapiens/Gene/Summary?db=core;g=BAX) |
| BIRC3 |  |  | CHMP4B |
| SIVA1 |  |  | IL1A |
| BID |  |  | CHMP3 |
| AIFM3 |  |  | IRF1 |
| DIABLO |  |  | CHMP2A |
| KRAS |  |  | TP53 |
| AIFM2 |  |  | CHMP7 |
| MOAP1 |  |  | CHMP4C |
| API5 |  |  | IRF2 |
| MAPK1 |  |  | CYCS |
| BAK1 |  |  | CHMP6 |
| CDKN1A |  |  | ELANE |
| BIRC7 |  |  | CHMP4A |
| AATK |  |  |  |
| FADD |  |  |  |
| NRAS |  |  |  |
| MAP3K5 |  |  |  |
| CCAR2 |  |  |  |
| CCAR1 |  |  |  |
| STAT3 |  |  |  |
| PEA15 |  |  |  |
| MAPK14 |  |  |  |
| RIPK1 |  |  |  |
| AVEN |  |  |  |
| AEN |  |  |  |
| CASP4 |  |  |  |
| BFAR |  |  |  |
| TP53AIP1 |  |  |  |
| AATF |  |  |  |
| TNFRSF1A |  |  |  |
| BBC3 |  |  |  |
| MYC |  |  |  |
| CIAPIN1 |  |  |  |
| NAIF1 |  |  |  |
| APAF1 |  |  |  |
| PERP |  |  |  |
| BAD |  |  |  |
| ANXA5 |  |  |  |
| TRIAP1 |  |  |  |
| AREL1 |  |  |  |
| ECSCR |  |  |  |
| TNFRSF10C |  |  |  |
| MDM2 |  |  |  |
| TNFRSF10D |  |  |  |
| CAAP1 |  |  |  |
| CASP14 |  |  |  |
| NFKB1 |  |  |  |
| JUN |  |  |  |
| TP53BP2 |  |  |  |
| TRAF2 |  |  |  |
| CASP5 |  |  |  |
| MTOR |  |  |  |
| BCL2L2 |  |  |  |
| E2F1 |  |  |  |
| ELAPOR1 |  |  |  |
| BIK |  |  |  |
| GZMB |  |  |  |
| DDIAS |  |  |  |
| PTEN |  |  |  |
| BLCAP |  |  |  |
| NIBAN2 |  |  |  |
| BCL2L14 |  |  |  |
| PYCARD |  |  |  |
| NIBAN1 |  |  |  |
| CDK1 |  |  |  |
| DFFA |  |  |  |
| EGFR |  |  |  |
| NOL3 |  |  |  |
| BECN1 |  |  |  |
| PTGS2 |  |  |  |
| MAPK3 |  |  |  |
| RAF1 |  |  |  |
| TRADD |  |  |  |
| ATM |  |  |  |
| ELAPOR2 |  |  |  |
| BCL2L10 |  |  |  |
| HSPA5 |  |  |  |
| FOXO3 |  |  |  |
| DAXX |  |  |  |
| HTRA2 |  |  |  |
| CDKN2A |  |  |  |
| AATBC |  |  |  |
| CCND1 |  |  |  |
| TET2 |  |  |  |
| TP73 |  |  |  |
| MAPK10 |  |  |  |
| TNFSF12 |  |  |  |
| TGFB1 |  |  |  |
| SEPTIN4 |  |  |  |
| TNFRSF25 |  |  |  |
| RELA |  |  |  |
| ACTA2 |  |  |  |
| CLU |  |  |  |
| BCL2L13 |  |  |  |
| HIF1A |  |  |  |
| MAPK9 |  |  |  |
| PIK3CG |  |  |  |
| VEGFA |  |  |  |
| SIRT1 |  |  |  |
| CSE1L |  |  |  |
| XAF1 |  |  |  |
| NIBAN3 |  |  |  |
| IGF1R |  |  |  |
| CDK2 |  |  |  |
| IGF1 |  |  |  |
| PMAIP1 |  |  |  |
| NFKBIA |  |  |  |
| VDAC1 |  |  |  |
| XIAPP3 |  |  |  |
| XIAPP2 |  |  |  |
| IL2 |  |  |  |
| HRK |  |  |  |
| DFFB |  |  |  |
| PPP1R13B |  |  |  |
| PDCD6 |  |  |  |
| RIPK3 |  |  |  |
| IFNG |  |  |  |
| DDIT3 |  |  |  |
| RNF7 |  |  |  |
| API5P1 |  |  |  |
| CTNNB1 |  |  |  |
| AKT2 |  |  |  |
| APP |  |  |  |
| STAT1 |  |  |  |
| CIAPIN1P |  |  |  |
| TRIAP1P1 |  |  |  |
| API5P2 |  |  |  |
| ENSG00000228998 |  |  |  |
| ENSG00000271253 |  |  |  |
| ENSG00000271623 |  |  |  |
| LOC100505995 |  |  |  |
| XIAPP1 |  |  |  |
| ENSG00000218073 |  |  |  |
| ENSG00000259286 |  |  |  |
| LOC100131159 |  |  |  |
| LOC390600 |  |  |  |
| ENSG00000237653 |  |  |  |
| ENSG00000288255 |  |  |  |
| LOC100129725 |  |  |  |
| LOC124903553 |  |  |  |
| BCL2A1 |  |  |  |
| PDCD5 |  |  |  |
| RASGRP1 |  |  |  |
| PPARG |  |  |  |
| MIR21 |  |  |  |
| ABL1 |  |  |  |
| PTK2 |  |  |  |
| TRAF1 |  |  |  |
| ESR1 |  |  |  |
| HSPB1 |  |  |  |
| THAP1 |  |  |  |
| IL6 |  |  |  |
| FAF1 |  |  |  |
| AKT3 |  |  |  |
| RB1 |  |  |  |
| IL1B |  |  |  |
| MAP2K1 |  |  |  |
| DAPK1 |  |  |  |
| RIPK2 |  |  |  |
| SYVN1 |  |  |  |
| GSK3B |  |  |  |
| BIRC8 |  |  |  |
| HMGB1 |  |  |  |
| CHUK |  |  |  |
| NFE2L2 |  |  |  |
| STK4 |  |  |  |
| SRC |  |  |  |
| PML |  |  |  |
| HRAS |  |  |  |
| CHEK2 |  |  |  |
| PIK3CA |  |  |  |
| CDKN1B |  |  |  |
| TERT |  |  |  |
| TNFRSF1B |  |  |  |
| PCNA |  |  |  |
| NGF |  |  |  |
| RNF34 |  |  |  |
| CD4 |  |  |  |
| TNFAIP3 |  |  |  |
| YWHAB |  |  |  |
| BIRC6 |  |  |  |
| IKBKB |  |  |  |
| TXN |  |  |  |
| NR3C1 |  |  |  |
| BRCA1 |  |  |  |
| MAP3K1 |  |  |  |
| PAK2 |  |  |  |
| NR4A1 |  |  |  |
| SOD1 |  |  |  |
| CAT |  |  |  |
| HSPA4 |  |  |  |
| ENDOG |  |  |  |
| CD40 |  |  |  |
| HMOX1 |  |  |  |
| JAK2 |  |  |  |
| EGF |  |  |  |
| CCNA2 |  |  |  |
| IRF1 |  |  |  |
| PRPF8 |  |  |  |
| SOD2 |  |  |  |
| TNFRSF11B |  |  |  |
| IL10 |  |  |  |
| HSP90AA1 |  |  |  |
| NLRP1 |  |  |  |
| IL3 |  |  |  |
| YWHAQ |  |  |  |
| CCNB1 |  |  |  |
| IFNA1 |  |  |  |
| IKBKG |  |  |  |
| MAP2K4 |  |  |  |
| CARD8 |  |  |  |
| TET2-AS1 |  |  |  |
| PDCD6IP |  |  |  |
| STK17B |  |  |  |
| IGFBP3 |  |  |  |
| CDK4 |  |  |  |
| IFI27 |  |  |  |
| HDAC9 |  |  |  |
| PRKCA |  |  |  |
| IL2RA |  |  |  |
| STK17A |  |  |  |
| CDK5 |  |  |  |
| AR |  |  |  |
| ERBB2 |  |  |  |
| YWHAE |  |  |  |
| FOS |  |  |  |
| SMPD1 |  |  |  |
| ATG5 |  |  |  |
| MAP3K7 |  |  |  |
| HIPK2 |  |  |  |
| PRKN |  |  |  |
| CTSD |  |  |  |
| KRT18 |  |  |  |
| NOS2 |  |  |  |
| TP63 |  |  |  |
| HDAC1 |  |  |  |
| SP1 |  |  |  |
| TNFRSF6B |  |  |  |
| TNFSF11 |  |  |  |
| CDH1 |  |  |  |
| ABCB1 |  |  |  |
| BNIP3 |  |  |  |
| ATR |  |  |  |
| PDCD4 |  |  |  |
| SQSTM1 |  |  |  |
| MADD |  |  |  |
| CASP12 |  |  |  |
| IER3IP1 |  |  |  |
| CSNK2A1 |  |  |  |
| DNM1L |  |  |  |
| RARA |  |  |  |
| DAPK3 |  |  |  |
| LGALS1 |  |  |  |
| YAP1 |  |  |  |
| HSPA1A |  |  |  |
| TNFSF13B |  |  |  |
| PDCD10 |  |  |  |
| PRKCE |  |  |  |
| IRF3 |  |  |  |
| BDNF |  |  |  |
| CD40LG |  |  |  |
| CXCR4 |  |  |  |
| YWHAZ |  |  |  |
| FAM215A |  |  |  |
| TIGAR |  |  |  |
| TGM2 |  |  |  |
| GAS2 |  |  |  |
| NGFR |  |  |  |
| PRF1 |  |  |  |
| FOXO1 |  |  |  |
| BAG1 |  |  |  |
| FLT3 |  |  |  |
| PIM1 |  |  |  |
| CXCL8 |  |  |  |
| PLK1 |  |  |  |
| FHIT |  |  |  |
| PIK3R1 |  |  |  |
| PTPA |  |  |  |
| IER3 |  |  |  |
| PIDD1 |  |  |  |
| PRKCI |  |  |  |
| STK11 |  |  |  |
| IL15 |  |  |  |
| IL24 |  |  |  |
| CHEK1 |  |  |  |
| GSN |  |  |  |
| CD27 |  |  |  |
| CARD16 |  |  |  |
| EP300 |  |  |  |
| WWOX |  |  |  |
| MMP9 |  |  |  |
| DNASE1L3 |  |  |  |
| PTPN13 |  |  |  |
| YWHAG |  |  |  |
| RPS3 |  |  |  |
| CRADD |  |  |  |
| JAK1 |  |  |  |
| TIA1 |  |  |  |
| PLEKHF1 |  |  |  |
| YWHAH |  |  |  |
| SPHK1 |  |  |  |
| STK3 |  |  |  |
| TRAF6 |  |  |  |
| H2AX |  |  |  |
| CDK6 |  |  |  |
| NOTCH1 |  |  |  |
| TNFRSF21 |  |  |  |
| ACIN1 |  |  |  |
| BMF |  |  |  |
| EIF2AK2 |  |  |  |
| TNFSF14 |  |  |  |
| ITPR1 |  |  |  |
| PPP1R13L |  |  |  |
| SHC1 |  |  |  |
| KIT |  |  |  |
| CIDEC |  |  |  |
| KDR |  |  |  |
| TLR2 |  |  |  |
| PRKCB |  |  |  |
| MIR34A |  |  |  |
| BMP6 |  |  |  |
| GDF15 |  |  |  |
| LGALS3 |  |  |  |
| TRAF3 |  |  |  |
| BCL10 |  |  |  |
| ATRAID |  |  |  |
| TLR4 |  |  |  |
| MIR146A |  |  |  |
| CIDEA |  |  |  |
| NPM1 |  |  |  |
| EIF2S1 |  |  |  |
| TNFRSF12A |  |  |  |
| TOP1.00 |  |  |  |
| IL5 |  |  |  |
| MAP3K14 |  |  |  |
| BCAP31 |  |  |  |
| FGF2 |  |  |  |
| GADD45A |  |  |  |
| ERN1.00 |  |  |  |
| CD5L |  |  |  |
| GAS5 |  |  |  |
| BRAF |  |  |  |
| NTRK1 |  |  |  |
| INS |  |  |  |
| STK24 |  |  |  |
| CREB1 |  |  |  |
| PPP1R15A |  |  |  |
| CDKN3 |  |  |  |
| MALAT1 |  |  |  |
| KAT5 |  |  |  |
| GAPDH |  |  |  |
| HGF |  |  |  |
| THOC1 |  |  |  |
| OLR1 |  |  |  |
| CENPS |  |  |  |
| FGFR3 |  |  |  |
| DNAJA3 |  |  |  |
| MET |  |  |  |
| BAG3 |  |  |  |
| UACA |  |  |  |
| BNIP3L |  |  |  |
| ICAM1 |  |  |  |
| CDC25C |  |  |  |
| DEDD |  |  |  |
| MT-RNR2 |  |  |  |
| ESR2 |  |  |  |
| CASP8AP2 |  |  |  |
| IL1A |  |  |  |
| CTSB |  |  |  |
| NQO1 |  |  |  |
| BAG6 |  |  |  |
| RPS6KB1 |  |  |  |
| SPP1 |  |  |  |
| REL |  |  |  |
| PDCD1 |  |  |  |
| IL4 |  |  |  |
| TSC2 |  |  |  |
| MAPK8IP1 |  |  |  |
| RBM5 |  |  |  |
| PCSK9 |  |  |  |
| RARB |  |  |  |
| VIM |  |  |  |
| NDRG1 |  |  |  |
| DAPK2 |  |  |  |
| PRKCZ |  |  |  |
| CENPS-CORT |  |  |  |
| PLEKHF2 |  |  |  |
| ATF2 |  |  |  |
| RNASEL |  |  |  |
| UNC5B |  |  |  |
| EPO |  |  |  |
| NFKB2 |  |  |  |
| EGR1 |  |  |  |
| OPA1 |  |  |  |
| LCK |  |  |  |
| FGFR2 |  |  |  |
| PTRH2 |  |  |  |
| MDM4 |  |  |  |
| SLC25A6 |  |  |  |
| STPG1 |  |  |  |
| HSPD1 |  |  |  |
| BAG4 |  |  |  |
| MAPK11 |  |  |  |
| FNIP2 |  |  |  |
| XBP1 |  |  |  |
| BCL2L12 |  |  |  |
| GORASP1 |  |  |  |
| SLC25A5 |  |  |  |
| DAD1 |  |  |  |
| BMP2 |  |  |  |
| MKI67 |  |  |  |
| MFN2 |  |  |  |
| ACHE |  |  |  |
| CXCL12 |  |  |  |
| ERBB4 |  |  |  |
| SUMO1 |  |  |  |
| STAT5A |  |  |  |
| PSEN1 |  |  |  |
| RASSF1 |  |  |  |
| AXIN1 |  |  |  |
| LMNB1 |  |  |  |
| FASN |  |  |  |
| SFRP1 |  |  |  |
| HSP90AB1 |  |  |  |
| MAP2K6 |  |  |  |
| BMP4 |  |  |  |
| CSF2 |  |  |  |
| PRKD1 |  |  |  |
| EZH2 |  |  |  |
| SIAH1 |  |  |  |
| FCMR |  |  |  |
| C1QBP |  |  |  |
| MEF2A |  |  |  |
| SLC25A4 |  |  |  |
| CAV1 |  |  |  |
| LYN |  |  |  |
| NOS3 |  |  |  |
| CDK11B |  |  |  |
| NLRP3 |  |  |  |
| SATB1 |  |  |  |
| KITLG |  |  |  |
| HSPA8 |  |  |  |
| BCL2L2-PABPN1 |  |  |  |
| ODC1 |  |  |  |
| SPHK2 |  |  |  |
| EIF2AK3 |  |  |  |
| BTK |  |  |  |
| MYCN |  |  |  |
| IRS1 |  |  |  |
| MAPT |  |  |  |
| ZC3H12A |  |  |  |
| SMAD7 |  |  |  |
| IL17A |  |  |  |
| LTBR |  |  |  |
| MEG3 |  |  |  |
| MALT1 |  |  |  |
| IFNB1 |  |  |  |
| AIMP1 |  |  |  |
| TXN2 |  |  |  |
| TSC1 |  |  |  |
| MAP2K7 |  |  |  |
| DPF2 |  |  |  |
| EI24 |  |  |  |
| PAK1 |  |  |  |
| FAIM2 |  |  |  |
| HK2 |  |  |  |
| RAC1 |  |  |  |
| LMNA |  |  |  |
| TAX1BP1 |  |  |  |
| TIAL1 |  |  |  |
| CSNK2A2 |  |  |  |
| UNC5A |  |  |  |
| NOX4 |  |  |  |
| MIR145 |  |  |  |
| SH3GLB1 |  |  |  |
| LCN2 |  |  |  |
| DAP3 |  |  |  |
| AURKA |  |  |  |
| IL9 |  |  |  |
| RET |  |  |  |
| ALOX12 |  |  |  |
| PHLDA1 |  |  |  |
| NTRK2 |  |  |  |
| PTK2B |  |  |  |
| CSF3 |  |  |  |
| HOTAIR |  |  |  |
| TRAF5 |  |  |  |
| BCL2L15 |  |  |  |
| RELB |  |  |  |
| GCG |  |  |  |
| CARD11 |  |  |  |
| SMAD2 |  |  |  |
| S100A9 |  |  |  |
| PPM1D |  |  |  |
| CD274 |  |  |  |
| IFIH1 |  |  |  |
| GSK3A |  |  |  |
| ANXA1 |  |  |  |
| DUSP19 |  |  |  |
| SPTAN1 |  |  |  |
| PTPN11 |  |  |  |
| TMBIM6 |  |  |  |
| TG |  |  |  |
| GIMAP5 |  |  |  |
| TNFRSF13B |  |  |  |
| CASP16P |  |  |  |
| CXCR3 |  |  |  |
| SET |  |  |  |
| PIK3CB |  |  |  |
| DYNLL1 |  |  |  |
| PHLPP1 |  |  |  |
| FAIM |  |  |  |
| GADD45B |  |  |  |
| CTLA4 |  |  |  |
| PINK1 |  |  |  |
| IGF2 |  |  |  |
| STAT5B |  |  |  |
| PDPK1 |  |  |  |
| HBB |  |  |  |
| CBL |  |  |  |
| PRKDC |  |  |  |
| DUSP1 |  |  |  |
| MLKL |  |  |  |
| THAP3 |  |  |  |
| PRKCQ |  |  |  |
| BCL6 |  |  |  |
| MEF2D |  |  |  |
| MMP2 |  |  |  |
| XRCC6 |  |  |  |
| CAPN1 |  |  |  |
| LEP |  |  |  |
| TNFSF13 |  |  |  |
| RHOB |  |  |  |
| HTT |  |  |  |
| ITGB1 |  |  |  |
| RFFL |  |  |  |
| CCND3 |  |  |  |
| CDC25A |  |  |  |
| HDAC2 |  |  |  |
| JUND |  |  |  |
| AXL |  |  |  |
| RAC2 |  |  |  |
| APC |  |  |  |
| ATF3 |  |  |  |
| PRKAA1 |  |  |  |
| CD28 |  |  |  |
| S100A8 |  |  |  |
| SH2D1A |  |  |  |
| TNFSF15 |  |  |  |
| GSDME |  |  |  |
| NUPR1 |  |  |  |
| ROCK1 |  |  |  |
| APOE |  |  |  |
| APEX1 |  |  |  |
| PPARA |  |  |  |
| THBS1 |  |  |  |
| TIMP1 |  |  |  |
| ING1 |  |  |  |
| IAPP |  |  |  |
| LGALS9 |  |  |  |
| TGFBR1 |  |  |  |
| ZFAT |  |  |  |
| LOC111255642 |  |  |  |
| CELF2 |  |  |  |
| PPP2CA |  |  |  |
| MIF |  |  |  |
| ASAH2 |  |  |  |
| PTPRC |  |  |  |
| ITGAM |  |  |  |
| TNFRSF8 |  |  |  |
| PPM1F |  |  |  |
| ERBB3 |  |  |  |
| PKN1 |  |  |  |
| CDK5R1 |  |  |  |
| SFN |  |  |  |
| BNIP1 |  |  |  |
| MLH1 |  |  |  |
| DNMT1 |  |  |  |
| PRKCH |  |  |  |
| MIR155 |  |  |  |
| DIDO1 |  |  |  |
| SFRP2 |  |  |  |
| TGFA |  |  |  |
| AGER |  |  |  |
| NOS1 |  |  |  |
| TYMP |  |  |  |
| LTA |  |  |  |
| PIK3R2 |  |  |  |
| MAP3K6 |  |  |  |
| MIR221 |  |  |  |
| TFAP2A |  |  |  |
| CCL2 |  |  |  |
| PRKAB1 |  |  |  |
| RHOA |  |  |  |
| FOXP3 |  |  |  |
| TIMP3 |  |  |  |
| IFI6 |  |  |  |
| SMPD2 |  |  |  |
| SERPINB5 |  |  |  |
| ATF4 |  |  |  |
| YY1 |  |  |  |
| PIM3 |  |  |  |
| PPIA |  |  |  |
| TSPO |  |  |  |
| SPATA4 |  |  |  |
| TLR3 |  |  |  |
| BCR |  |  |  |
| NEAT1 |  |  |  |
| SFRP4 |  |  |  |
| ATF6 |  |  |  |
| MAVS |  |  |  |
| PLK3 |  |  |  |
| LATS2 |  |  |  |
| TGFBR2 |  |  |  |
| EIF5A |  |  |  |
| GDNF |  |  |  |
| PLSCR1 |  |  |  |
| TFRC |  |  |  |
| RNF144B |  |  |  |
| SRGN |  |  |  |
| CTSG |  |  |  |
| IL13 |  |  |  |
| HAX1 |  |  |  |
| SOX4 |  |  |  |
| RARS1 |  |  |  |
| IL18 |  |  |  |
| HNRNPA1 |  |  |  |
| HDAC3 |  |  |  |
| TFPT |  |  |  |
| CDK11A |  |  |  |
| PIM2 |  |  |  |
| GAS6 |  |  |  |
| RACK1 |  |  |  |
| NOD1 |  |  |  |
| TAF6 |  |  |  |
| SIRT3 |  |  |  |
| FIS1 |  |  |  |
| CAPN2 |  |  |  |
| DNAJB13 |  |  |  |
| DAB2IP |  |  |  |
| THAP2 |  |  |  |
| RPS6KA1 |  |  |  |
| MIR29B1 |  |  |  |
| ID1 |  |  |  |
| NRG1 |  |  |  |
| ITGB3BP |  |  |  |
| PRDX1 |  |  |  |
| PRNP |  |  |  |
| NR1H2 |  |  |  |
| TNFRSF9 |  |  |  |
| SKP2 |  |  |  |
| SMAD4 |  |  |  |
| PTGES |  |  |  |
| CD8A |  |  |  |
| KMT2A |  |  |  |
| TUG1 |  |  |  |
| CD44 |  |  |  |
| CXCL10 |  |  |  |
| RASSF6 |  |  |  |
| P2RX7 |  |  |  |
| VDAC2 |  |  |  |
| RAG1 |  |  |  |
| DNASE1 |  |  |  |
| WT1 |  |  |  |
| ITCH |  |  |  |
| CD36 |  |  |  |
| SOCS3 |  |  |  |
| KLF11 |  |  |  |
| ETS1 |  |  |  |
| NRP1 |  |  |  |
| MIR29A |  |  |  |
| PARK7 |  |  |  |
| SAV1 |  |  |  |
| GRB2 |  |  |  |
| MYB |  |  |  |
| EAF2 |  |  |  |
| TRIM28 |  |  |  |
| ITGB4 |  |  |  |
| GSTP1 |  |  |  |
| ABCC1 |  |  |  |
| SNCA |  |  |  |
| RPS27A |  |  |  |
| MAX |  |  |  |
| HSP90B1 |  |  |  |
| ALOX5 |  |  |  |
| MIR214 |  |  |  |
| JAK3 |  |  |  |
| PDGFRB |  |  |  |
| DEDD2 |  |  |  |
| MAP2K3 |  |  |  |
| PLCG1 |  |  |  |
| PVT1 |  |  |  |
| PRDX2 |  |  |  |
| USP7 |  |  |  |
| PSIP1 |  |  |  |
| HSF1 |  |  |  |
| CREBBP |  |  |  |
| IL7 |  |  |  |
| TRIM27 |  |  |  |
| RUNX2 |  |  |  |
| AHR |  |  |  |
| PPP1CA |  |  |  |
| PDCD2 |  |  |  |
| EPHA3 |  |  |  |
| ANGPT2 |  |  |  |
| CCND2 |  |  |  |
| NLRC4 |  |  |  |
| HUWE1 |  |  |  |
| TUBB |  |  |  |
| FEM1B |  |  |  |
| CCN4 |  |  |  |
| PTHLH |  |  |  |
| HMGCR |  |  |  |
| TNFRSF4 |  |  |  |
| SGK1 |  |  |  |
| NUMA1 |  |  |  |
| DCC |  |  |  |
| MPO |  |  |  |
| RALA |  |  |  |
| PIR |  |  |  |
| BEX3 |  |  |  |
| MXD1 |  |  |  |
| CCN1 |  |  |  |
| BUB1B |  |  |  |
| EIF4G2 |  |  |  |
| CD19 |  |  |  |
| SYK |  |  |  |
| TMBIM4 |  |  |  |
| UBC |  |  |  |
| EPHA2 |  |  |  |
| MAPK13 |  |  |  |
| AURKB |  |  |  |
| MAP2K2 |  |  |  |
| CIDEB |  |  |  |
| PECAM1 |  |  |  |
| DKK1.00 |  |  |  |
| F2R |  |  |  |
| KLLN |  |  |  |
| GLI1 |  |  |  |
| ETS2 |  |  |  |
| MAGEH1 |  |  |  |
| ING3 |  |  |  |
| HNRNPK |  |  |  |
| NTN1 |  |  |  |
| SFRP5 |  |  |  |
| PKM |  |  |  |
| MIR143 |  |  |  |
| UBD |  |  |  |
| VDR |  |  |  |
| RALBP1 |  |  |  |
| PLA2G6 |  |  |  |
| LRBA |  |  |  |
| PLSCR3 |  |  |  |
| FYN |  |  |  |
| CD47 |  |  |  |
| E2F3 |  |  |  |
| UCA1 |  |  |  |
| TPT1 |  |  |  |
| NLRP10 |  |  |  |
| CAPNS1 |  |  |  |
| GABARAP |  |  |  |
| PRKACA |  |  |  |
| OSMR |  |  |  |
| THPO |  |  |  |
| TYMS |  |  |  |
| MAP3K15 |  |  |  |
| MMP1 |  |  |  |
| NOD2 |  |  |  |
| CSF1 |  |  |  |
| WEE1 |  |  |  |
| UNC5D |  |  |  |
| ACP1 |  |  |  |
| TICAM1 |  |  |  |
| HBEGF |  |  |  |
| RNF13 |  |  |  |
| MIR222 |  |  |  |
| PRKCG |  |  |  |
| IKBKE |  |  |  |
| MSH2 |  |  |  |
| ADIPOQ |  |  |  |
| IGF2R |  |  |  |
| PEG3 |  |  |  |
| MIR195 |  |  |  |
| MAGED1 |  |  |  |
| NFKBIB |  |  |  |
| CRP |  |  |  |
| EIF4E |  |  |  |
| GRAMD4 |  |  |  |
| CUL1 |  |  |  |
| MIR15A |  |  |  |
| PDCD7 |  |  |  |
| MAF |  |  |  |
| SFPQ |  |  |  |
| TFDP1 |  |  |  |
| NME6 |  |  |  |
| PPP2R2B |  |  |  |
| PRDX5 |  |  |  |
| AGAP2 |  |  |  |
| PLAUR |  |  |  |
| MIR126 |  |  |  |
| ADA |  |  |  |
| TGFB2 |  |  |  |
| GZMA |  |  |  |
| TRAF4 |  |  |  |
| PROM1 |  |  |  |
| CALR |  |  |  |
| ABCG2 |  |  |  |
| TNFRSF11A |  |  |  |
| SHISA5 |  |  |  |
| RXRA |  |  |  |
| PIN1 |  |  |  |
| MECOM |  |  |  |
| JADE1 |  |  |  |
| PPM1L |  |  |  |
| PTPN6 |  |  |  |
| BMI1 |  |  |  |
| TP73-AS1 |  |  |  |
| CAST |  |  |  |
| HSPA9 |  |  |  |
| G6PD |  |  |  |
| TUBA1B |  |  |  |
| BCAR1 |  |  |  |
| SH3KBP1 |  |  |  |
| PRL |  |  |  |
| BEX2 |  |  |  |
| CTSL |  |  |  |
| SMAD3 |  |  |  |
| CYLD |  |  |  |
| HSD17B10 |  |  |  |
| EPOR |  |  |  |
| BMX |  |  |  |
| PARM1 |  |  |  |
| TNFAIP8 |  |  |  |
| DNTT |  |  |  |
| MEFV |  |  |  |
| PSMC6 |  |  |  |
| SMPD3 |  |  |  |
| PHB1 |  |  |  |
| RAD21 |  |  |  |
| PTGS1 |  |  |  |
| UBE2K |  |  |  |
| INPP5D |  |  |  |
| MIR125A |  |  |  |
| FGFR1 |  |  |  |
| FOXM1 |  |  |  |
| PIK3C3 |  |  |  |
| ELAVL1 |  |  |  |
| TWIST1 |  |  |  |
| DBNL |  |  |  |
| PSMA2 |  |  |  |
| PSMA4 |  |  |  |
| H1-3 |  |  |  |
| ITGA5 |  |  |  |
| PRKAR1B |  |  |  |
| IRAK1 |  |  |  |
| CRYBA1 |  |  |  |
| DYNLL2 |  |  |  |
| PSMD1 |  |  |  |
| PSMD9 |  |  |  |
| PRKAR2B |  |  |  |
| CLSPN |  |  |  |
| APIP |  |  |  |
| H1-5 |  |  |  |
| CHP1 |  |  |  |
| PSMC5 |  |  |  |
| EXOG |  |  |  |
| UBB |  |  |  |
| TFDP2 |  |  |  |
| MYD88 |  |  |  |
| FNTA |  |  |  |
| PKP1 |  |  |  |
| CEACAM6 |  |  |  |
| DSP |  |  |  |
| TLE1 |  |  |  |
| CHP2 |  |  |  |
| PSMC4 |  |  |  |
| PSMB2 |  |  |  |
| PPP3CB |  |  |  |
| PSMD13 |  |  |  |
| PSMD8 |  |  |  |
| PRKAR1A |  |  |  |
| PSMA3 |  |  |  |
| IRAK2 |  |  |  |
| PSMA1 |  |  |  |
| STK26 |  |  |  |
| PIK3R5 |  |  |  |
| PSMB11 |  |  |  |
| PSMB7 |  |  |  |
| NMT1 |  |  |  |
| TJP2 |  |  |  |
| H1-0 |  |  |  |
| PRKX |  |  |  |
| PPP3R1 |  |  |  |
| OCLN |  |  |  |
| SEM1 |  |  |  |
| PPP3CA |  |  |  |
| PSMD7 |  |  |  |
| IRAK3 |  |  |  |
| PPP3R2 |  |  |  |
| PRKAR2A |  |  |  |
| PSMC2 |  |  |  |
| BRMS1 |  |  |  |
| PSMC3 |  |  |  |
| IL3RA |  |  |  |
| ANKRD13C |  |  |  |
| ADD1 |  |  |  |
| PSMD2 |  |  |  |
| PSMA8 |  |  |  |
| PSMD12 |  |  |  |
| PSMB3 |  |  |  |
| CEACAM5 |  |  |  |
| PSMB1 |  |  |  |
| PSMA6 |  |  |  |
| H1-1 |  |  |  |
| TICAM2 |  |  |  |
| IL1RAP |  |  |  |
| PSMB6 |  |  |  |
| TJP1 |  |  |  |
| KPNB1 |  |  |  |
| PRKACB |  |  |  |
| PSMD6 |  |  |  |
| PSMD3 |  |  |  |
| DSG1 |  |  |  |
| ENDOD1 |  |  |  |
| PSME3 |  |  |  |
| PSMB8 |  |  |  |
| PSMD11 |  |  |  |
| PIK3CD |  |  |  |
| PSME2 |  |  |  |
| UBA52 |  |  |  |
| IRAK4 |  |  |  |
| OMA1 |  |  |  |
| PSMA7 |  |  |  |
| ARHGAP10 |  |  |  |
| PSMB10 |  |  |  |
| SNAI2 |  |  |  |
| PSMB4 |  |  |  |
| PLEC |  |  |  |
| APPL1 |  |  |  |
| PSMB5 |  |  |  |
| PSMD10 |  |  |  |
| DSG2 |  |  |  |
| LY96 |  |  |  |
| SIK1 |  |  |  |
| IL1R1 |  |  |  |
| PDK4 |  |  |  |
| PSMA5 |  |  |  |
| MYBBP1A |  |  |  |
| KPNA1 |  |  |  |
| PSMC1 |  |  |  |
| PRKACG |  |  |  |
| TLE5 |  |  |  |
| PSME4 |  |  |  |
| HMGB2 |  |  |  |
| H1-4 |  |  |  |
| CSF2RB |  |  |  |
| PSMD4 |  |  |  |
| PSME1 |  |  |  |
| PSMD14 |  |  |  |
| H1-2 |  |  |  |
| DSG3 |  |  |  |
| PSMD5 |  |  |  |
| PSMB9 |  |  |  |
| PPP3CC |  |  |  |
| PSMF1 |  |  |  |
| ZNF304 |  |  |  |
| PIK3R3 |  |  |  |
| CD14 |  |  |  |

**Supplementary Table 2.** Primers

| **Gene** | **Sequence (5’ to 3’)** | **Use** |
| --- | --- | --- |
| **HUMAN** | | |
| UNC5D Fw | TGAACTGCAGATGCCATAGG | qPCR |
| UNC5D Rv | GGTTTCAGGGACACTGTGGT | qPCR |
| GAPDH Fw | GGACGTGCAGGGCAACTACC | qPCR |
| GAPDH Rv | AGCCGACGATGAGAAAGGGG | qPCR |
